# TRIF Regulates BIC/miR-155 via the ERK Signaling Pathway to Control the ox-LDL-Induced Macrophage Inflammatory Response

**DOI:** 10.1155/2018/6249085

**Published:** 2018-06-07

**Authors:** Yaxi Wu, Jinshan Ye, Ruiwei Guo, Xing Liang, Lixia Yang

**Affiliations:** ^1^Department of Cardiology, Kunming General Hospital of Chengdu Military Area, Yunnan 650032, China; ^2^Department of Postgraduate, Kunming Medical University, Yunnan 650032, China; ^3^Institution of Cardiovascular Research, Xinqiao Hospital, Third Military Medical University, Chongqing 400037, China

## Abstract

Toll/IL-1R-domain-containing adaptor-inducing IFN-*β* (TRIF) is an important adaptor for TLR3- and TLR4-mediated inflammatory signaling pathways. Recent studies have shown that TRIF plays a key role in vessel inflammation and atherosclerosis; however, the precise mechanisms are unclear. We investigated the mechanisms of the TRIF-regulated inflammatory response in RAW264.7 macrophages under oxidized low-density lipoprotein (ox-LDL) stimulation. Our data show that ox-LDL induces TRIF, miR-155, and BIC expression, activates the ERK_1/2_ and SOCS1-STAT3-NF-*κ*B signaling pathways, and elevates the levels of IL-6 and TNF-*α* in RAW264.7 cells. Knockdown of TRIF using TRIF siRNA suppressed BIC, miR-155, IL-6, and TNF-*α* expression and inhibited the ERK_1/2_ and SOCS1-STAT3-NF-*κ*B signaling pathways. Inhibition of ERK_1/2_ signaling also suppressed BIC and miR-155 expression. These findings suggest that TRIF plays an important role in regulating the ox-LDL-induced macrophage inflammatory response and that TRIF modulates the expression of BIC/miR-155 and the downstream SOCS1-STAT3-NF-*κ*B signaling pathway via ERK_1/2_. Therefore, TRIF might be a novel therapeutic target for atherosclerosis.

## 1. Introduction

Atherosclerosis (AS) is a chronic arterial disease and a major threat to public health worldwide, as it is a main cause of cardiovascular disease (CVD), ischemic stroke, and local thrombosis [1]. AS is now recognized as a chronic inflammatory disorder that is induced by oxidized low-density lipoprotein (ox-LDL) accumulation and inflammation in the arterial intima under hypercholesterolemic conditions [2]. Multiple cells, such as macrophages, lymphocytes, endothelial cells, and smooth muscle cells, contribute to the occurrence and development of AS [3]. Macrophages play especially important roles in this pathophysiological process, as they are the major effector cells that stimulate the vascular inflammatory response through various inflammatory mediators and form foam cells in atherosclerotic lesions, thereby promoting plaque formation and impacting plaque stability [4, 5]. Therefore, it is important to explore novel mechanisms underlying the ox-LDL-induced macrophage inflammatory response.

Toll/IL-1R-domain-containing adaptor-inducing IFN-*β* (TRIF) is a Toll/IL-1R- (TIR-) domain-containing adaptor [6, 7]. TRIF plays a pivotal role following the activation of Toll-like receptor (TLR) 3 and 4 signaling, leading to the production of inflammatory mediators through the activation of several transcription factors, including NF-*κ*B, IRF3, and AP-1 [8–11]. Vorkapic E et al. showed that knockout of TRIF suppressed angiotensin (Ang) II-induced aneurysm formation and vascular inflammation *in vivo* [12]. Lundberg and colleagues reported that TRIF deficiency in hematopoietic cells reduced atherogenic diet-induced vascular inflammation and protected against atherosclerosis, as shown in Ldlr^−/−^ mice after receiving a bone marrow transfer from TRIF-deficient mice [13]. Another study revealed that Ldlr^−/−^ mice with a loss-of-function mutation in TRIF (Lps2) were significantly protected from atherosclerosis and exhibited reduced cytokine secretion from peritoneal macrophages under hyperlipidemic conditions [14]. Overall, the above studies indicate that TRIF plays a key role in vessel inflammation and atherosclerosis; however, its precise mechanism is unclear.

MicroRNA (miRNA) is an endogenous, short length (~22 nucleotides) noncoding RNA. Recently, several reports have shown that miRNAs, especially miR-155, play a pivotal role in the regulation of inflammatory responses in AS by binding to the 3′-untranslated region (UTR) of target mRNAs [15]. There was a previous report by Tian et al. that miR-155 was upregulated in macrophages by ox-LDL stimulation. They also reported that miR-155 is involved in ox-LDL-induced macrophage inflammatory response, including expression of inflammatory factors IL-6 and TNF-*α*. Other studies have also demonstrated that elevated miR-155 promotes foam cell formation and atherosclerosis by repressing its downstream target genes, including Bcl-6, SOCS1, HMG box-transcription protein 1 (HBP1), and mitogen-activated protein kinase 10 (MAP3K10) [16–19]. Although ox-LDL-induced miR-155 play a key role for AS occurrence and progress, the potential mechanism is still unknown.

In the present study, we investigated the potential mechanism of ox-LDL-induced miR-155 and inflammation response in macrophages and found that ox-LDL induced TRIF expression and activated ERK_1/2_ signal, then enhanced the expression of B-cell integration cluster (BIC, miR-155 host gene)/miR-155, thus promoting inflammation mediator production.

## 2. Materials and Methods

### 2.1. Materials

Raw264.7 macrophages were purchased from CellBio (Shanghai, China). Oxidized low-density lipoprotein (ox-LDL) was purchased from Peking Union-Biology Co. Ltd. (Beijing, China). TriPure Isolation Reagent, X-tremeGENE siRNA Transfection Reagent, X-tremeGENE HP DNA Transfection Reagent, the Transcriptor First Strand cDNA Synthesis Kit, and FastStart Universal SYBR Green Master Mix were purchased from Roche (Switzerland). The following primary antibodies were used in this study: rabbit antisuppressor of cytokine signaling 1 (SOCS1) (Abcam, UK), rabbit antiphosphorylation-signal transducer and activator of transcription 3 (p-STAT3) (Cell Signaling Technology, USA), rabbit antiphosphorylated-protein kinase (MAPK)/extracellular signal-regulated kinase_1/2_ (p-ERK_1/2_) and ERK_1/2_ (Cell Signaling Technology, USA), NF*κ*B p65 (Cell Signaling Technology, USA), and rabbit anti-*β*-actin (Cell Signaling Technology, USA). Horseradish peroxidase- (HRP-) conjugated AffiniPure goat anti-rabbit IgG was purchased from Beijing Zhongshan Golden Bridge Biotechnology Co. Ltd. (Beijing, China). NF-*κ*B luciferase reporter plasmid (pNF*κ*B-luc) was purchased from Beyotime Inc. (Jiangsu, China). High-glucose Dulbecco's Modified Eagle Medium (DMEM) and fetal bovine serum (FBS) were purchased from Gibco (USA). ERK inhibitor, SCH772984, was purchased from MedChem Express (USA). TRIF siRNA sequences have been designed and synthesized by GenePharma Co. Ltd. (Shanghai, China). All other reagents were commercially available and used as received.

### 2.2. Cell Culture and Treatment

RAW264.7 cells were cultured in DMEM with 10% FBS and 1% penicillin/streptomycin. These cells were treated with 20 *μ*g/mL ox-LDL for 0, 6, 12, and 24 h and then underwent further study.

### 2.3. Transfection with TRIF siRNA

TRIF siRNA and negative control (NC) had been transfected into RAW264.7 cells as previously described [20]. The sequences of TRIF siRNA are the following: (1) 5′-GCU AUG UAA CAC ACC GCU GTT-3′; (2) 5′-GGA CAU ACG UUA CAC UCC ACC AACA GTT-3′; (3) 5′-GGU CAA ACG UGA CAC UCa ACC UGC GTT-3′; and NC sequence: 5′-ACG UGA CAC GUU CGG AGA ATT-3′.

### 2.4. RT-qPCR Analysis of miR-155 and B-Cell Integration Cluster (BIC), IL-6, and TNF-*α* mRNA Expression

Total RNA was isolated from treated RAW264.7 cells, and cDNA was synthesized using a Transcriptor First Strand cDNA Synthesis Kit according to the manufacturer's instructions. The following primers were used in the quantitative PCR (qPCR) assay: miR-155 forward primer, 5′-ACACTCCAGCTGGGTTAATGCTAATCGTG-3′, miR-155 reverse primer, 5′-CTCAACTGGTGTCGTGGAGT-3′; U6 forward primer, 5′-GTGCTCGCTTCGGCAGCA-3a′, U6 reverse primer, 5′-CAAAATATGGAACGCTTC-3′; BIC forward primer, 5′-CAAACCAGGAAGGGGAAGTGT-3′, BIC reverse primer, 5′-TAGGAGTCAGTCAGAGGCCAA-3′; TRIF forward primer, 5′-GCAGGCAGCACAAGTACAAC-3′, TRIF reverse primer, 5′-GTGCTCGGTTTCAGGCAATG-3′; TNF-*α* forward primer, 5′-GACCCTCACACTCAG ATCATC-3′, TNF-*α* reverse primer: 5′-GAACCTGGGAGTAGATAAGG; IL-6 forward primer, 5′-GTATGAACAACGATGATGCACTTG3′, IL-6 reverse primer, 5′-ATGGTACTCCAGAAGACCAGAGGA-3′; and *β*-actin forward primer, 5′-CACGGCATCGTCACCAACT-3′, *β*-actin reverse primer, 5′-GTCCTACGGAAAACGGCAGA-3′. PCR amplification was performed under the following conditions: 95°C for 10 min followed by 95°C for 30 s, 60°C for 30 s, and 72°C for 30 s for 35 cycles. Data analysis was performed using the 2^−△△CT^ method to determine the relative level of target gene expression, which was normalized to U6 or *β*-actin expression.

### 2.5. Evaluation of TRIF, SOCS1, p-STAT3, NF*κ*B p65, p-ERK_1/2_, and ERK_1/2_ Protein Levels by Western Blot

Western blot was performed as previously described [21]. Briefly, total protein was isolated from treated cells. Each protein sample was separated by 10% SDS-PAGE and transferred onto polyvinylidene difluoride (PVDF) membranes. PVDF membranes were then incubated with antibodies, including TRIF, SOCS1, p-STAT3, p-ERK_1/2_, and ERK_1/2_ antibodies. The protein bands were detected by an enhanced chemiluminescence detection system.

### 2.6. Analysis of NF-*κ*B Promoter Activity by Dual-Luciferase Reporter Assay

RAW264.7 cells were seeded onto 24-well tissue culture plates and cultured overnight. The cells were cotransfected with 100 ng of pNF*κ*B-luc and 100 ng of pRL-TK as a control using X-tremeGENE HP DNA Transfection Reagent for 6 h. Then, TRIF siRNA or negative control (NC) was transfected into these cells. After 24 h, the cell culture media were replaced with DMEM containing 10% FBS, and the cells were treated with 20 *μ*g/mL ox-LDL for 24 h. The treated cells were harvested in the lysis buffer, and luciferase activity was analyzed [22].

### 2.7. Statistical Analysis

Data are presented as the mean ± S.E. Statistical comparisons among groups were performed using one-way ANOVA. *p* < 0.05 was considered statistically significant.

## 3. Results

### 3.1. ox-LDL Induced TRIF Expression in a Time-Dependent Manner

**Figure 1:**
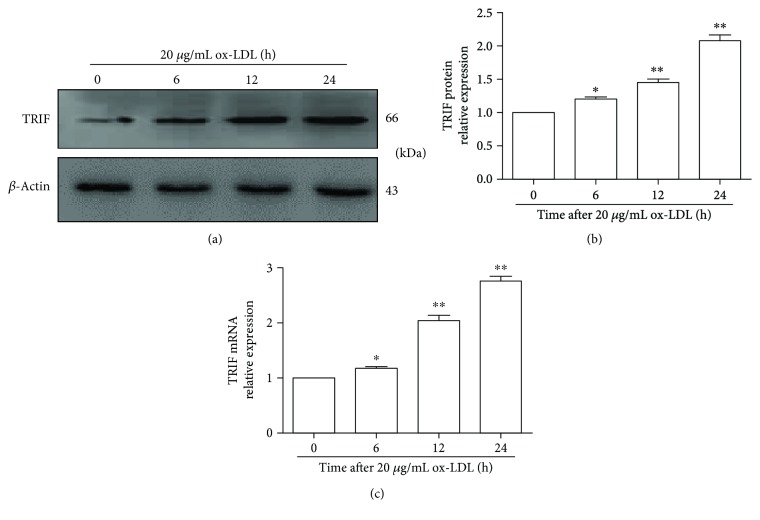
Effect of ox-LDL on TRIF expression in RAW264.7 cells. (a) TRIF protein expression was evaluated by Western blot. RAW264.7 cells were treated with 20 *μ*g/mL ox-LDL for the indicated times. Representative bands show the expression of TRIF protein (upper panel) and *β*-actin protein (lower panel). (b) Histograms illustrate TRIF protein expressions, which were normalized to *β*-actin expression. The data represent the mean ± S.E. of three independent experiments. (c) qPCR was used to detect the level of TRIF mRNA. The data represent the mean ± S.E. of four independent experiments. ^∗^*p* < 0.05 and ^∗∗^*p* < 0.01 versus the 0 h time point.

To explore the effect of TRIF on the ox-LDL-induced inflammatory macrophage inflammatory response, we investigated the expression of TRIF protein after RAW264.7 cells were treated with 20 *μ*g/mL ox-LDL for 24 h. As shown in Figures 1(a) and 1(b), TRIF protein expressions were significantly increased at 6, 12, and 24 h compared to those at 0 h (*p* < 0.05). Moreover, the level of TRIF mRNA was higher at 6, 12, and 24 h than at 0 h (*p* < 0.05). Therefore, ox-LDL increased TRIF levels in a time-dependent manner. These data showed that ox-LDL gradually increased the expression of TRIF protein and mRNA over time, suggesting that TRIF might be involved in the ox-LDL-induced inflammatory response.

### 3.2. ox-LDL Induced miR-155 Expression and the Macrophage Inflammatory Response

**Figure 2:**
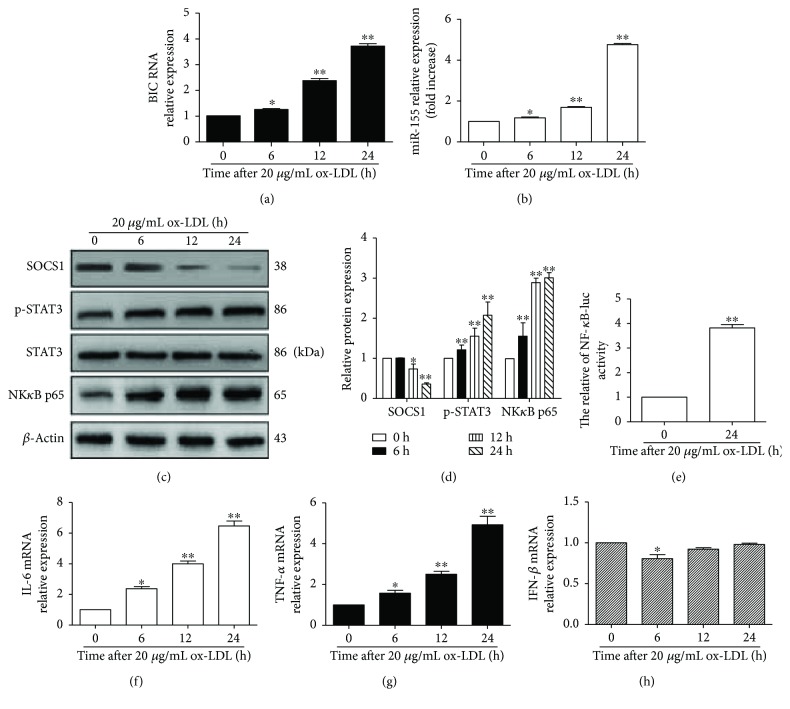
ox-LDL induced miR-155 generation and the macrophage inflammatory response. RAW264.7 cells were treated with 20 *μ*g/mL ox-LDL for the indicated times. (a and b) qPCR was used to detect the expression of miR-155HG (BIC) and miR-155 in macrophages. (c) SOCS1, NF*κ*B p65, STAT3, and p-STAT3 protein expression was evaluated using Western blot. Representative bands show the expression of SOCS1 protein (upper panel), p-STAT3 (second panel), STAT3 (third panel), NF*κ*B p65 (fourth panel), and *β*-actin protein (lower panel). (d) Histograms illustrated the SOCS1, NF*κ*B p65, and p-STAT3 protein expressions, which were normalized to *β*-actin expression. The data represented the mean ± S.E. of three independent experiments. (e) The promoter activity of NF-*κ*B was analyzed by dual-luciferase reporter assay. (f, g, h) The expressions of IL-6, TNF-*α*, and IFN-*β* mRNA were measured using qPCR. The data represent the mean ± S.E. of four independent experiments. ^∗^*p* < 0.05 and ^∗∗^*p* < 0.01 versus the 0 h time point.

In this study, we analyzed the expression of miR-155 and BIC following the exposure of RAW264.7 cells to ox-LDL. These data demonstrated that the expression of BIC RNA was markedly increased at 6, 12, and 24 h compared to that at 0 h (*p* < 0.05) (Figure 2(a)). Similarly, the expression of miR-155 increased with prolonged ox-LDL treatment time compared to that at the 0 h time point (*p* < 0.05) (Figure 2(b)). We further studied the effect of ox-LDL on the miR-155-mediated inflammatory signaling pathway. These data showed that the level of SOCS1 protein, a target of miR-155, was lower at 6, 12, and 24 h after ox-LDL exposure than at 0 h (*p* < 0.05). In contrast, the expression of p-STAT3 was significantly elevated after the RAW264.7 cells were treated with ox-LDL for 6, 12, and 24 h compared to the 0 h time point (*p* < 0.05). The expression of NF*κ*B p65 protein was similar with p-STAT3. (Figures 2(c) and 2(d)). NF-*κ*B promoter activity and the level of IL-6 and TNF-*α* mRNA expression were also increased when RAW264.7 cells were exposed to 20 *μ*g/mL ox-LDL (Figures 2(e)–2(g)). Additionally, the expression of IFN-*β* mRNA was lower at 6 h than at 0 h (*p* < 0.05), and the IFN-*β* mRNA expression at 12 h and 24 h was not significantly different compared to that at 0 h (*p* > 0.05) (Figure 2(h)). These data suggested that ox-LDL not only induced miR-155 generation but also activated the inflammatory signaling pathway via miR-155.

### 3.3. Inhibition of TRIF by TRIF siRNA Suppressed ox-LDL-Induced miR-155 Generation

**Figure 3:**
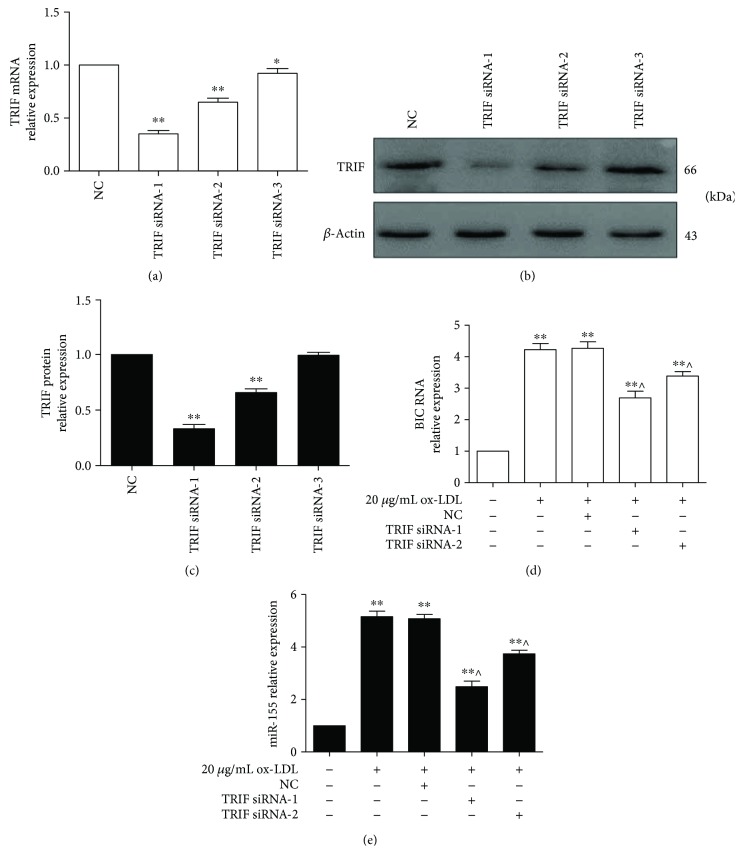
TRIF silencing suppressed BIC and miR-155 expression. (a and b) TRIF mRNA and protein expression was inhibited by TRIF siRNA. Three TRIF siRNA oligos and negative control (NC) were separately transfected into RAW264.7 cells for 24 h, and then, the cells were harvested for detection of TRIF mRNA and protein expressions by qPCR and Western blot, respectively. Representative bands in B show the expression of TRIF protein (upper panel) and *β*-actin protein (lower panel). (c) Histograms illustrate the TRIF protein expressions, which were normalized to *β*-actin expression. The data represent the mean ± S.E. of three independent experiments. ^∗^*p* < 0.05 and ^∗∗^*p* < 0.01 versus the NC group. (d and e) BIC RNA and miR-155 expression was evaluated by qPCR. RAW264.7 cells were preincubated with TRIF siRNA-1 or siRNA-2 for 24 h followed by treatment with 20 *μ*g/mL ox-LDL for 24 h. The data represent the mean ± S.E. of four independent experiments. ^∗∗^*p* < 0.01 versus the control group and ^∧^*p* < 0.05 versus the 20 *μ*g/mL ox-LDL/NC group.

To explore the role of TRIF in ox-LDL-induced inflammation, we silenced TRIF mRNA and protein using TRIF siRNA. The data showed that the three TRIF siRNA oligos markedly suppressed TRIF mRNA and protein expression (*p* < 0.05) and that the TRIF siRNA-1 and siRNA-2 oligo was more effective in silencing TRIF expression than the other siRNA oligos (Figures 3(a)–3(c)). Interestingly, the expression of BIC RNA was significantly decreased when the Raw264.7 cells were preincubated with TRIF siRNA-1 and siRNA-2, then exposed to ox-LDL compared to cells that were preincubated with NC followed by ox-LDL exposure (*p* < 0.05) (Figure 3(d)). Similarly, miR-155 expression was lower in the ox-LDL/TRIF siRNA group than that in the ox-LDL/NC group (*p* < 0.05) (Figure 3(e)). However, the levels of BIC and miR-155 remained higher in the ox-LDL/TRIF siRNA group than those in the control group (*p* < 0.05). These data demonstrated that TRIF knockdown could partly reverse ox-LDL-induced miR-155 generation and suggested that TRIF modulates miR-155 generation. Additionally, given to TRIF siRNA-1 was better to suppress TRIF expression; therefore, it was used to knock down TRIF expression in subsequent experiments.

### 3.4. TRIF Silencing Inactivated the miR-155-Mediated Inflammatory Pathway

**Figure 4:**
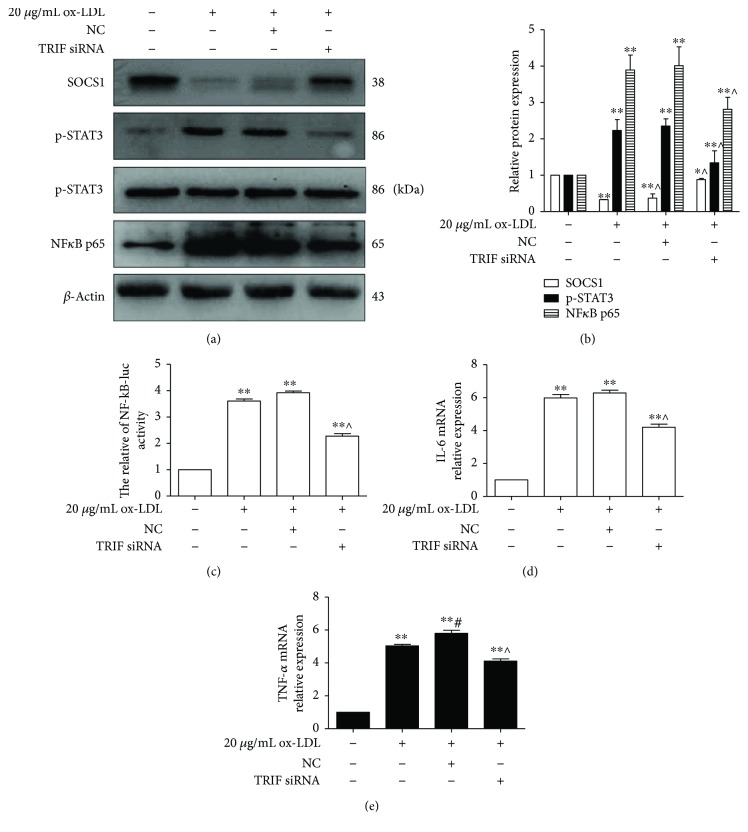
Knockdown of TRIF suppressed the miR-155-mediated inflammatory pathway. RAW264.7 cells were preincubated with TRIF siRNA for 24 h followed by treatment with 20 *μ*g/mL ox-LDL for 24 h. (a) The levels of SOCS1 NF*κ*B p65, STAT3, and p-STAT3 protein were evaluated using Western blot. Representative bands showed the levels of SOCS1 protein (upper panel), p-STAT3 (second panel), STAT3 (third panel), NF*κ*B p65 (fourth panel), and *β*-actin protein (lower panel). (b) Histograms illustrated the SOCS1, NF*κ*B p65, and p-STAT3 protein expressions, which were normalized to *β*-actin expression. (c) NF-*κ*B promoter activity was analyzed using a dual-luciferase reporter assay. (d and e) The expressions of IL-6 and TNF-*α* mRNA were measured using qPCR. The data represent the mean ± S.E. of four independent experiments. ^∗^*p* < 0.05 versus the control group, ^∗∗^*p* < 0.01 versus the control group, ^#^*p* < 0.05 versus the 20 *μ*g/mL ox-LDL group, and ^∧^*p* < 0.05 versus the 20 *μ*g/mL ox-LDL/NC group.

TRIF plays an important role in the MyD88-independent inflammatory pathway. Given that TRIF could upregulate the generation of miR-155, we hypothesized that TRIF knockdown could suppress the miR-155-mediated inflammatory pathway. Our data demonstrated that the level of SOCS1 protein expression was decreased after RAW264.7 cells were challenged with ox-LDL in the ox-LDL/NC group and partly restored in the ox-LDL/TRIF siRNA group. In contrast to the expression of p-STAT, NF*κ*B p65 was significantly inhibited in the ox-LDL/TRIF siRNA group compared to that in the ox-LDL/NC group (*p* < 0.05) (Figures 4(a) and 4(b)). The promoter activity of NF-*κ*B was higher in the ox-LDL, ox-LDL/NC, and ox-LDL/TRIF siRNA groups than that in the control group (*p* < 0.05). Moreover, NF-*κ*B activity was notably suppressed in the ox-LDL/TRIF siRNA group compared to that in the ox-LDL/NC group (*p* < 0.05) (Figure 4(c)). Meanwhile, the expressions of IL-6 and TNF-*α* mRNA were consistent with the NF-*κ*B promoter activity (Figures 4(d) and 4(e)). These data revealed that TRIF knockdown inactivated the miR-155-mediated inflammatory pathway, suggesting that TRIF was a novel and important target for the inhibition of the ox-LDL-induced macrophage inflammatory response.

### 3.5. ERK_1/2_ Signaling Is Involved in TRIF-Mediated miR-155 Generation

**Figure 5:**
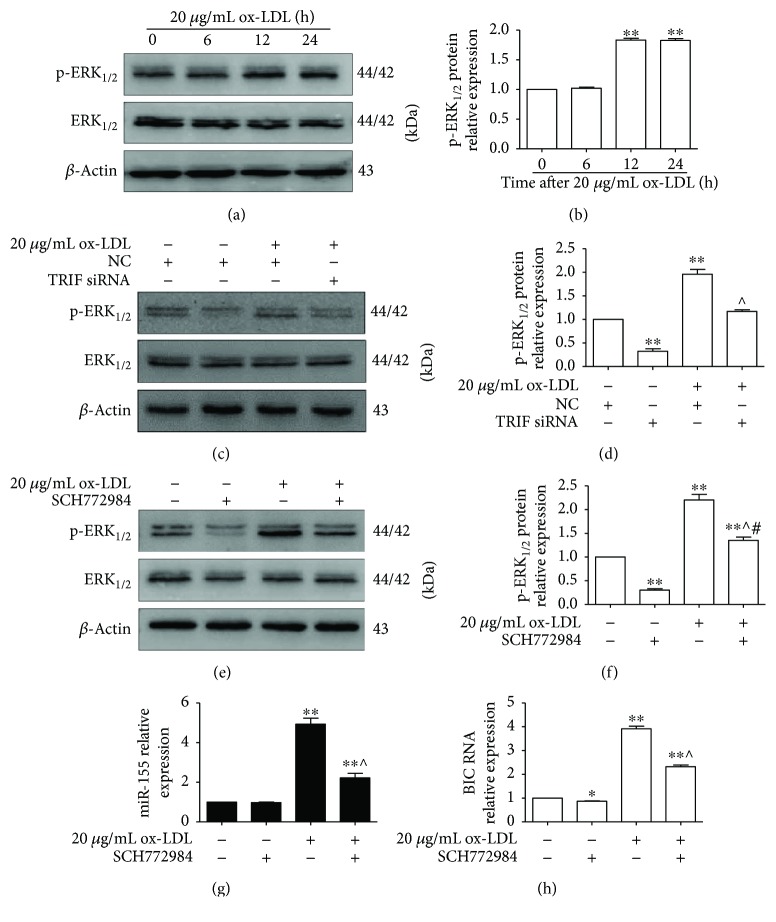
ERK_1/2_ is involved in the regulation of BIC and miR-155 expression by TRIF. (a) The expression of p-ERK_1/2_ protein was evaluated by Western blot. RAW264.7 cells were treated with 20 *μ*g/mL ox-LDL for the indicated times. Representative bands showed the expression of p-ERK_1/2_ protein (upper panel), p-ERK_1/2_ protein (middle panel), and *β*-actin protein (lower panel). (b) Histograms illustrated the p-ERK_1/2_ protein level, which was normalized to the ERK_1/2_ level. The data represented the mean ± S.E. of three independent experiments. ^∗^*p* < 0.05 and ^∗∗^*p* < 0.01 versus the 0 h time point. (c) TRIF silencing suppressed p-ERK_1/2_ expression. RAW264.7 cells were transfected with NC or TRIF siRNA for 24 h followed by treatment with 20 *μ*g/mL ox-LDL for 24 h. Representative bands show the expression of p-ERK_1/2_ protein (upper panel), p-ERK_1/2_ protein (middle panel), and *β*-actin protein (lower panel). (d) Histograms illustrate the p-ERK_1/2_ protein expression, which was normalized to the ERK_1/2_ expression. The data represent the mean ± S.E. of three independent experiments. ^∗∗^*p* < 0.01 versus the NC group and ^∧^*p* < 0.05 versus the TRIF siRNA/ox-LDL group. (e) The level of p-ERK_1/2_ expression was suppressed by SCH772984 (ERK_1/2_ inhibitor). RAW264.7 cells were preincubated with 1 *μ*M SCH772984 for 2 h followed by treatment with 20 *μ*g/mL ox-LDL for 24 h. (f) Histograms illustrate the p-ERK_1/2_ protein expression, which was normalized to the ERK_1/2_ expression. (g and h) The expressions of miR-155 and BIC RNA were measured using qPCR. RAW264.7 cells were preincubated with 1 *μ*M SCH772984 for 2 h followed by treatment with 20 *μ*g/mL ox-LDL for 24 h. The data represent the mean ± S.E. of three independent experiments. ^∗∗^*p* < 0.01 versus the control group, ^∧^*p* < 0.05 versus the 20 *μ*g/mL ox-LDL group, and ^#^*p* < 0.05 versus the SCH772984 group.

ERK_1/2_ signaling plays a key role in the pathophysiology of AS [23–25]. In our study, we investigated the role of ERK_1/2_ in ox-LDL-induced miR-155 generation in RAW264.7 cells. As shown in Figures 5(a) and 5(b), the level of p-ERK_1/2_ was elevated at 12 and 24 h after macrophages were treated with 20 *μ*g/mL ox-LDL compared to that at the 0 h time point (*p* < 0.05). Subsequent experiments showed that TRIF silencing by TRIF siRNA significantly inhibited the expression of p-ERK_1/2_ compared to the NC group (*p* < 0.05). Furthermore, the level of p-ERK_1/2_ expression was lower in the TRIF siRNA/ox-LDL group than that in the ox-LDL group (*p* < 0.05) (Figures 5(c) and 5(d)). Moreover, the level of p-ERK_1/2_ expression was lower in the SCH772984 group than that in the control group (*p* < 0.05) and lower in the ox-LDL/SCH772984 group than that in the ox-LDL group (*p* < 0.05) (Figures 5(e) and 5(f)). Additionally, the expressions of BIC and miR-155 were suppressed in the ox-LDL/SCH772984 group compared with that in the ox-LDL group (*p* < 0.05) (Figures 5(g) and 5(h)). These data suggest that ERK_1/2_ signaling is involved in TRIF-mediated miR-155 generation.

## 4. Discussion

Herein, we determined that ox-LDL induced TRIF, miR-155, and BIC (the precursor of miR-155) expression. Knockdown of TRIF expression partly reversed ox-LDL-induced BIC and miR-155 expression, inactivated the miR-155-modulated SOCS1-STAT3-NF-*κ*B pathway, and reduced the production of inflammatory mediators. Moreover, we showed that ERK_1/2_ signaling is involved in the induction of mediated miR-155 generation by TRIF. These data suggest that TRIF promotes the ox-LDL-induced macrophage inflammatory response by inducing miR-155 generation.

Recent studies have shown that TRIF plays a critical role in modulating the progression of AS and vessel inflammation in animal models [13, 14] following the activation of TLR 3 and 4 in the endosomes [26]. Our data showed that ox-LDL induced the expression of TRIF in macrophages; furthermore, activation of NK-*κ*B signaling and upregulated TNF-*α* and IL-6 expression were also been found [21, 27]. While knockdown of TRIF using TRIF siRNA partly suppressed ox-LDL-induced NK-*κ*B activation and inflammatory mediator expression, our data suggest that upregulation of TRIF by ox-LDL promotes the macrophage inflammatory response and that TRIF is a novel target for blocking inflammatory mediator release from macrophages. Additionally, the expression of IFN-*β* was decreased when RAW264.7 cells were exposed to ox-LDL for 6 h and then gradually restored to baseline. This finding might be due to the induction of TRAF family member-associated NF-*κ*B activator (TANK) monoubiquitination by ox-LDL and the subsequent suppression of TRIF-dependent IFN-*β* expression [28].

BIC was first identified in avian leukosis virus-induced B lymphomagenesis as a collaborator with c-myc even though it is a nonprotein-coding RNA due to the lack of a large open reading frame (ORF) [29–31]. Lagos-Quintana et al. identified the miR-155 foldback precursor sequence within a conserved region of BIC [32]. Eis and colleagues subsequently found that the level of miR-155 expression was dependent on the level of BIC RNA and the regulation of pre-miR-155 generation [33]. Recently, O'Connell et al. demonstrated that activation of TLRs by their ligands upregulated miR-155 through either a MyD88-dependent or MyD88-independent (TRIF) signaling pathway in THP-1 cells. Moreover, BIC RNA was shown to be involved in TLR ligand-induced miR-155 expression [34]. Similarly, our study found that ox-LDL induced BIC and miR-155 expression in RAW264.7 cells, thereby suppressing the target gene SOCS1 and activating the STAT3-NF-*κ*B signaling pathway. Knockdown of TRIF suppressed the expression of BIC and miR-155, partly inactivated the STAT3-NF-*κ*B signaling pathway, and downregulated the expression of inflammatory mediators, thus suggesting that TRIF induces miR-155 expression and activates its downstream signaling pathways by regulating the level of BIC RNA.

It is well known that activation of the ERK_1/2_ signaling pathway plays an important role in controlling several cellular biological responses, including cell cycle arrest, cell survival, and apoptosis [35–37]. Recently, studies have shown that ERK_1/2_ activation is involved in ox-LDL-induced endothelial cell injury [38–40], vascular smooth muscle cell (VSMC) proliferation and migration [41], and the macrophage inflammatory response [42, 43]. Consistent with the above research, our study also demonstrated that ERK_1/2_ signaling pathways are activated in macrophages following stimulation with ox-LDL. Interestingly, we found that knockdown of TRIF hampered the ox-LDL-mediated activation of ERK_1/2_ signaling pathways, suggesting that activation of ERK_1/2_ signaling is dependent on TRIF when macrophages are exposed to ox-LDL. Luan et al. showed that knockdown of TRIF-related adaptor molecule (TRAM) using TRAM siRNA suppressed Broncho-Vaxom-induced ERK_1/2_ activation in RAW264.7 cells [44]. Other studies have revealed that lipopolysaccharide (LPS) induces ERK_1/2_ activation through TRIF and MyD88- and TRIF-dependent signaling *in vivo* and *in vitro* [45–47]. According to the results of the above research, activation of ERK_1/2_ might be at least partly dependent on TRIF signaling when macrophages are stimulated by ox-LDL. In addition, our results suggest that ERK_1/2_ plays an important role in the regulation of BIC and miR-155 expression. Our findings are consistent with those of the previous report that the activation of ERK_1/2_ and c-Jun N-terminal kinase (JNK) pathways could upregulate BIC and miR-155 expression [48]. However, the exact mechanism requires additional investigation.

In conclusion, these findings illustrate that TRIF pathways play an important role in the regulation of the ox-LDL-induced macrophage inflammatory response. The elevation in TRIF leads to ERK_1/2_ signal activation, which in turn enhances the expression of BIC/miR-155, thus promoting inflammation mediator production. This study precisely defined an important mechanism underlying the ox-LDL-induced macrophage inflammatory response and showed that TRIF is a novel therapeutic target for atherosclerosis.
